# A transdisciplinary framework for managing metabolic dysfunction associated steatotic liver disease

**DOI:** 10.3389/fphar.2026.1767844

**Published:** 2026-02-24

**Authors:** Sania Kouser, Sanketh V. Sharma, Arun Bhanu, Subrahmanya Kumar Kukkupuni, Chethala N. Vishnuprasad

**Affiliations:** 1 Ayurveda Biology and Holistic Nutrition, The University of Transdisciplinary Health Sciences and Technology (TDU), Bengaluru, Karnataka, India; 2 National Institute of Advanced Studies (NIAS), Indian Institute of Science campus, Bengaluru, Karnataka, India

**Keywords:** *Ayurveda*, MASLD, precision medicine, transdisciplinary, treatment

## Abstract

Metabolic dysfunction-associated steatotic liver disease (MASLD) is rapidly emerging as a public health issue across the globe. Its complex etiopathology and association with diverse comorbidities poses significant challenges to conventional pharmacological drugs derived from the “*lock-and-key*” paradigm of pharmacology. To overcome the challenges of complexity and non-linear dynamics underlying the disease biology of MASLD, this article proposes a transdisciplinary framework combining holistic disease management principles of traditional medicines with molecular precision of modern biomedicine. *Ayurveda* - an exceptionally designed and widely practiced Indian Systems of Medicine (ISM) - is explored as a representative model for this framework. This article outlines a unified, biologically plausible transdisciplinary approach incorporating dual-diagnostic workflows, integrative decision interface, and stage-adaptive management algorithm for prevention, early intervention, and advanced disease care. The framework emphasizes integrative and personalized strategies integrating nutrition, lifestyle modification and drug interventions, harnessing the molecular precision of biomedicine alongside the systemic effects of phytochemical diversity. The transdisciplinary model seeks to shift the focus from ‘disease treatment’ to ‘health restoration’ and long-term wellness. The article highlights the scopes and challenges of this framework to offer more comprehensive, sustainable, and patient-centred solutions for MASLD.

## 1 Introduction

The rising global prevalence of non-communicable diseases (NCDs), and associated mortality and reduced quality and expectancy of life, poses one of the most pressing public health challenges of the 21st century (Hunter and Reddy, 2013; World Health Organization, 2014; World Health Organization, 2025). Modifiable and non-modifiable risk factors such as unhealthy diet, physical inactivity, globalization-induced lifestyle changes, aging, stress, metabolic abnormalities, tobacco and alcohol use, as well as genetic and environmental factors are fuelling the prevalence of NCDs (Cuevas Garciá-Dorado et al., 2019; Brauer et al., 2024; Formichi et al., 2025). Metabolic dysfunction-associated steatotic liver disease (MASLD), driven primarily by dysregulated glucose and lipid metabolism, is one such emerging global health issue (Feng et al., 2024; Scoditti et al., 2024). The multifactorial aetiology and systemic pathophysiology of MASLD often constrain the effectiveness of conventional molecular medicines, making an integrative medicine approach, effectively merging holistic and reductionist views of biology, relevant and imperative. This perspective article underscores the urgency of creating a transdisciplinary knowledge framework, bridging epistemologically and ontologically different medical systems, for an omniscient view of the MASLD disease biology; and examines its potential benefits, opportunities, and challenges in advancing MASLD management within the evolving landscape of NCDs.

### 1.1 MASLD - the “silent epidemic”: disease trajectory and global trends

MASLD is a spectrum of liver disorders ranging from simple steatosis to progressive metabolic dysfunction-associated steatohepatitis (MASH), fibrosis, cirrhosis, and MASH-associated hepatocellular carcinoma (HCC) (Tacke et al., 2024). As a cause and consequence of systemic metabolic imbalance, MASLD is characterized by excessive triglyceride accumulation within hepatocytes, accompanied by at least one cardiometabolic risk factor such as hypertension, overweight, increased plasma triglycerides, reduced plasma HDL-cholesterol and type 2 diabetes (T2D) (Rinella et al., 2023). The condition was first described in 1980 as *non-alcoholic steatohepatitis* (NASH) to distinguish from alcohol-induced hepatic steatosis. It was renamed as *non-alcoholic fatty liver disease* (NAFLD) in 1986, and later redefined as *metabolic dysfunction-associated fatty liver disease* (MAFLD) to capture a broader clinical spectrum linked to obesity or T2D or both (Ludwig et al., 1980; Schaffner and Thaler, 1986; Eslam et al., 2020). In 2023, a multi-society Delphi consensus among three major liver associations in Europe, North America, and Latin America adopted the term MASLD, aligning the nomenclature with its complex pathophysiology and metabolic determinants (Rinella et al., 2023).

A recent meta-analysis on MASLD reports a global prevalence of 32.4%, projected to reach 55.4% by 2040, posing substantial clinical and economic burdens (Nguyen et al., 2022; Riazi et al., 2022). While the prevalence is highest in Latin America (44.4%) and lowest in Western Europe (25.1%), a rapid increase in obesity and T2D in regions like Middle East and North Africa (MENA), and Asia drives these global statistics (Younossi et al., 2023, 2024a; 2024b). In India, the prevalence is much higher than the global average (68.2%) and corroborates with India’s status as “global capital of diabetes” (Mohan et al., 2025). The growing global prevalence of MASLD, the observed regional disparities, and its bidirectional association with overweight, obesity and T2D draws urgent attention to MASLD as a public health issue comparable to other epidemics and emphasize the need of tailored region-specific and personalized management strategies (Quek et al., 2023; Zargar et al., 2025). Noticeably India is the first country to include MASLD into the National Programme for Prevention and Control of Cancer, Diabetes, Cardiovascular Diseases and Stroke (NPCDCS) which aims at tackling the NCDs (Prasad et al., 2023).

### 1.2 The multisystem pathophysiology of MASLD is centred around impaired glucose and lipid metabolism

The disease trajectory and global trends of MASLD reveal two key aspects; its complex multisystem consequences and strong association with impaired glucose and lipid metabolism (Sandireddy et al., 2024; Targher et al., 2024; Steinberg et al., 2025). In normal physiology, dietary glucose is transported to the liver for glycogen storage or energy production, while insulin released from pancreatic β-cells suppresses hepatic gluconeogenesis and stimulates glucose uptake into muscle and adipose tissue. Both the liver and adipose together regulate the synthesis, storage, and breakdown of lipids. Excess glucose reaching these tissues will undergo *de novo lipogenesis* (DNL) and stored as triglycerides. During the fed-fasting cycle, the body efficiently switches between glucose and fat as the primary energy source through insulin-regulated coordination of lipolysis and gluconeogenesis between adipose, liver and muscle. Disruptions of this ‘metabolic flexibility’ due to behavioural, physiological and psychological factors is a key driver of NCDs like MASLD (Sanders and Griffin, 2015; Adeva-Andany et al., 2016; Luo and Liu, 2016; Cho et al., 2023).

**FIGURE 1:**
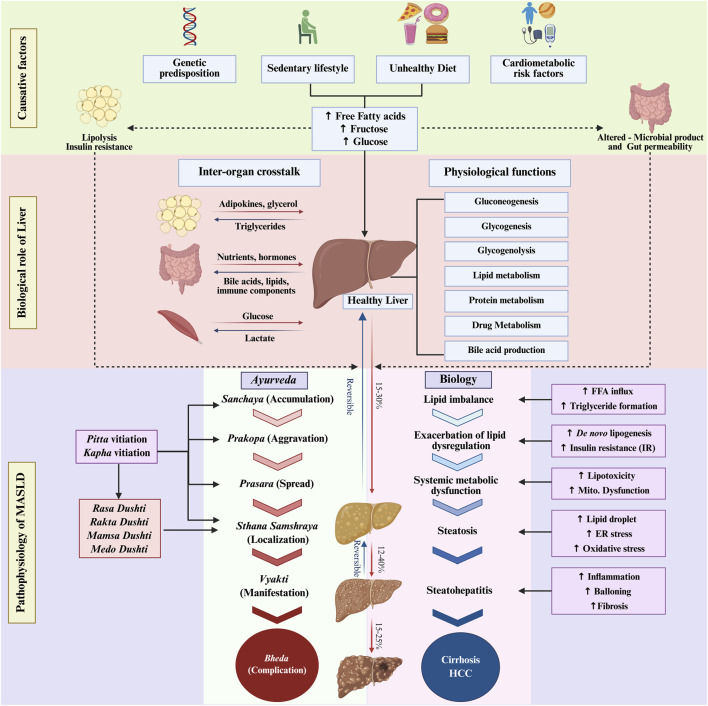
Liver biology and pathophysiology. Schematic representation of the healthy liver illustrating its core physiological functions, inter-organ crosstalk and the etiological drivers that accelerate MASLD progression. The lower purple panel presents a biologically plausible correlation of Ayurvedic concepts of disease progression with molecular events. Parallels drawn are theoretical correlations rather than as linear or definitive equivalences. Created in BioRender. Kouser, S. (2026) https://BioRender.com/ck1d2w9.

In MASLD, diets rich in carbohydrates over-activate transcription factors like carbohydrate-responsive element-binding protein (ChREBP) and sterol regulatory element-binding protein-1c (SREBP-1c), increasing hepatic DNL and triglyceride synthesis, leading to lipid overload, peripheral insulin resistance (IR), hyperinsulinemia and hyperglycaemia (Gastaldelli and Cusi, 2019). IR in liver further increases gluconeogenesis and DNL, while muscle and adipose tissue become resistant to insulin-mediated glucose uptake. Also, the increase in lipolysis in the adipose tissue releases excess free fatty acids (FFAs) into circulation, overburdening hepatic β-oxidation and causing accumulation of triglycerides, diacylglycerol and ceramides ultimately leading to hepatic steatosis (Trauner et al., 2010; Gastaldelli and Cusi, 2019; Kahn et al., 2019; Merz and Thurmond, 2020; Ahmed et al., 2021). All these biochemical changes progressively disrupt glucose and lipid homeostasis, which is a hallmark of the body’s metabolic dysfunction and MASLD pathogenesis (Bril et al., 2014). Chronic low-grade inflammation, recognised as a consequence of obesity induced IR, and lipotoxicity triggered release of nuclear factor-κB (NF-κB) and pro-inflammatory markers such as tumour necrosis factor-alpha (TNF-α) and interleukin-1β (IL-1β) are also known to contribute to the pathogenesis of MASLD, liver injury and fibrosis (Rohm et al., 2022; Dong et al., 2024) (Figure 1).

Among the extrahepatic consequences of MASLD, cardiovascular diseases (CVDs) are the leading cause of mortality due to pro-atherogenic lipid profile resulting from endothelial dysfunction, dyslipidaemia, increased VLDL secretion, and reduced LDL clearance (Villanova et al., 2005; Lawler et al., 2021; Younossi et al., 2023). Malignancies like pancreatic, colorectal, stomach, lung, breast, ovarian and prostate cancers represent the second most common death (Mantovani et al., 2022b; Azimi et al., 2025; Issa et al., 2025; Kogiso et al., 2025). MASLD also predicts the onset and progression of chronic kidney disease (CKD); shows a bidirectional relationship with muscle protein metabolism causing sarcopenia; and intrinsically connected with endocrine disorders like polycystic ovary syndrome (PCOS) and hypothyroidism (Bhanji et al., 2017; Mantovani et al., 2022a; Hong et al., 2023; Hutchison et al., 2023; Bilson et al., 2024; Li et al., 2024; Xu et al., 2024). These diverse diseases reflect the multidirectional systemic crosstalk in MASLD involving a shared metabolic milieu of obesity, hypertension, insulin resistance and mitochondrial dysfunction and proinflammatory cytokines, primarily driven by impaired glucose and lipid metabolism (Sandireddy et al., 2024).

## 2 Diagnosis and treatment of MASLD: challenges and the imperative for a paradigm shift

The complex pathophysiology, asymptomatic early stages, lack of consensus on standard diagnostic protocols, absence of universally accepted pharmacological interventions, and strong association with multiple comorbidities make early diagnosis and effective management of MASLD a great challenge (Ma et al., 2025; Adinolfi et al., 2026). Although non-invasive and invasive diagnostic options like biomarkers, imaging techniques and liver biopsy exist, their limited accessibility, affordability and patient compliance restrict widespread use (Garg et al., 2025; Nowak et al., 2025). MASLD diagnosis is often incidental during routine health check-ups as most of the early-stage symptoms like mild fatigue, bloating and abdominal discomfort are non-specific and often overlooked. Sometimes even the advanced stages like fibrosis, cirrhosis and cancer remain with non-specific symptoms or asymptomatic making MASLD underdiagnosed until serious outcomes (cirrhosis or HCC) occur (Sharma and Arora, 2020; Mishra et al., 2022).

Management approaches for MASLD can be broadly categorized into preventive, therapeutic and surgical interventions. Although lifestyle and dietary modifications remain as the cornerstone of MASLD prevention, poor patient adherence often limits long-term benefits (Arora et al., 2021; Wang et al., 2023). For instance, while the Mediterranean diet is beneficial for MASLD management, its global adoption is limited due to cultural, geographic, seasonal, and socioeconomic differences (Tsofliou et al., 2022; Cueto-Galán et al., 2025). Similarly, aerobic exercise and resistance training for weight loss is limited by resources, supervision, and difficulty in sustaining significant weight loss (Willis et al., 2012; Mahmood et al., 2023).

The major challenge in therapeutics is the absence of FDA-approved drugs for MASLD or cirrhosis (Mallet et al., 2024; Drygalski and Drygalski, 2025). Resmetirom (a thyroid hormone receptor beta (THR-β) agonist), the first FDA-approved drug for MASH and Saroglitazor (a dual PPARα/γ agonist), approved in India for T2D and MASH treatment are promising drug options, but needs more data on sustained histological benefits and long-term safety (Chhabra et al., 2022; Chaudhuri et al., 2023; Harrison et al., 2024; Mir et al., 2024).

Other pharmacological targets like incretin hormone agonists (Semaglutide and Trizeptide), SGLT2 inhibitors, FGF-21 analogue, PPAR agonists, antioxidants, and anti-inflammatory agents are actively investigated for MASLD management, but none have demonstrated clinically significant improvement in liver outcomes across diverse patient populations (Musso et al., 2017; Francque et al., 2021; Loomba et al., 2023, 2024; Sanyal et al., 2025). Surgical approaches including Roux-en-Y gastric bypass and liver transplantation, forms the third strategy. Though bariatric surgery and endoscopic bariatric therapies are beneficial to some extent, patients with end stage cirrhosis are generally left with liver transplantation as the only solution and it is expensive and associated with higher rates of complications, malnutrition, sarcopenia, morbidity and mortality. They often require multi-speciality care including hepatologists, endocrinologists, cardiologists, and dietitians (Fakhry et al., 2019; Lassailly et al., 2020; Aminian et al., 2021). The perpetually growing prevalence and strong association with co-morbid conditions makes a *one-size-fits-all* approach irrelevant for MASLD diagnosis and management.

Are current clinical frameworks for MASLD overlooking something essential? Probably ‘YES’. Philosophically the epistemology and ontology of modern biomedicine is grounded in molecular reductionism emphasizing a *lock-and-key* model of disease mechanisms and drug discovery. While this approach has been highly effective in treating infectious diseases, its limitations in managing complex lifestyle disorders like MASLD are increasingly evident. Its non-linear dynamics, multisystem pathophysiology and the role of systemic metabolic dysfunction in the pathogenesis demands integration of molecular and systemic perspectives for early diagnosis, prevention, and therapy. Resonating with the global interest in complementary and alternative medicine (CAM), the MASLD management landscape calls for an integrative framework that incorporates holistic principles of disease biology from complementary systems (World health organization, 2023). Indian Systems of Medicine (ISMs) like *Ayurveda* and Traditional Chinese Medicine (TCM) are driving transformative progress in this direction. Of the two, *Ayurveda* stands out as the oldest codified medical system deeply rooted in the Indian philosophical axioms of logic, epistemology and metaphysics.

## 3 The liver and MASLD in *Ayurveda* - distinctive understanding of its biology and pathophysiology

*Ayurveda*, a holistic system of medicine originating from the Indian subcontinent, offers a distinct epistemology and ontology for understanding the biological mechanisms underlying human health and disease. This unique worldview, however, presents challenges when attempting to integrate it with contemporary scientific discourse. The following section provides a brief overview of how *Ayurveda* understands the liver, its biological functions and the disease biology. A glossary is provided separately for readers to understand the unique *Sanskrit* terminologies used in *Ayurveda*.

In *Ayurveda*, the liver is referred to by the term *Yakrit*, a *Sanskrit* word meaning “to govern” (Apte, 1890). The description of *Yakrit* highlights its principal and crucial role in modulating *“Agni” -* the metabolic fire responsible for digestion, metabolism, and nutrient transformation (Sastry, 1997e). In *Ayurveda* parlance, after digestion, the food (*Ahara*) is transformed into an absorbable form referred to as “*Ahara Rasa*” which then transforms into “*Rasa Dhatu*,” the basic element of nourishment that circulates throughout the body (Sastry, 1997b; Murthy, 2001a). Upon reaching *Yakrit* (liver), *Rasa Dhatu* transforms into “*Rakta Dhatu*” (whole blood), producing “*Pitta*” (∼ bile juice) as a byproduct that get secreted into the gastro-intestinal tract (GIT) to support digestion, metabolism and nutrient transformation (Acharya, 1992a; Sastry, 1997f; Sastry, 1997d; Sastry, 1997a; Murthy, 2001b; Murthy, 2001c).

Although Ayurvedic narrative of liver biology does not map directly to the contemporary biological understanding of hepatology and the disease construct of MASLD, as a science that studies biological changes, *Ayurveda* offers a structured explanation of disease pathophysiology through a unique “*six-stage disease progression model*” referred to as *Shad-Kriyakala* (Chaple et al., 2016; Chauhan et al., 2017; Bishnoi et al., 2024). This framework centres around a few core concepts of *Ayurveda*, central to the biology of health and disease: *Ama*, *Agni*, *Kapha* and *Pitta*. *Ama* represents the accumulated ‘metabolic waste’ in the body arising from improper and incomplete nutrient metabolism. *Agni* refers to the digestive and metabolic functions responsible for preventing *Ama* accumulation (Sastry, 1997c; Naaz et al., 2025). *Kapha* and *Pitta* constitute two of the three fundamental biological forces (*Dosha*) that govern the physiological and biochemical homeostasis. Drawing from this model in *Ayurveda*, this article proposes an *Ayurveda-Biology hypothesis* of MASLD progression, with plausible correlation of Ayurvedic concepts of disease progression with molecular events (Figure 1). However, this correlation requires more detailed research for deeper scientific validation. *Ayurveda* posits that the MASLD progression begins when inappropriate diet and lifestyle - particularly those increasing *Kapha* and *Pitta* - impair *Agni* functions and lead to *Ama* accumulation (*Sanchaya* stage). Excess consumption of sweet, sour, savour, oily and heavy to digest foods are typically known to impair *Dosha* (*Kaptha* and *Pitta*) and *Agni* functions at GIT. The vitiated *Dosha* and accumulated *Ama* disrupts the dynamic equilibrium of physiological and biochemical functions in different parts of the body (*Prakopa* stage) (Asija and Singh, 2016; Saini and Lata, 2025). Eventually the absorbable form of food (*Ahara Rasa*) and nourishment (*Rasa Dhatu*) get vitiated, but circulate in the body and reach *Yakrit,* the liver (*Prasara* stage) (Acharya, 1992b; Murthy, 2001d). A simple analogy can illustrate this phenomenon: If the body is viewed as a house, each organ is a room with a defined function. When the accumulated waste in one room remains undisposed, it decomposes and eventually affects the entire house. At the liver, the pathophysiology is localized (*Sthana Samshraya* stage) causing liver steatosis, which in turn manifests with various clinical symptoms (*Vyakti* stage) and ends-up with complications (*Bheda* stage).

The key insight from this perspective is the foundational role of the gut in systemic health. An earlier work from Thottapillil and colleagues highlighted gut as a convergence point for an integrative Ayurveda-Biology framework of diabetes management; which holds true for MASLD as well (Thottapillil et al., 2021). Dietary quality and quantity, eating behaviour, seasonal and circadian rhythms, physical activity, and mental stress are key determinants of gut functions that influence nutrient transformation and metabolic balance. From an Ayurvedic perspective, impaired digestive–metabolic capacity (*Agni-mandya*) and accumulation of *Ama* create an internal milieu that can be conceptually aligned to inflammation and gut-microbiome dysbiosis resulting in compromised intestinal barrier function and ‘leaky-gut’ effect (Mishra et al., 2025; Shruti et al., 2025). This permits microbes, their products and other toxins to reach the liver, eventually triggering inflammation and oxidative stress culminating in MASLD and cirrhosis (Ray, 2015; Portincasa et al., 2021). Therefore, the emphasis *Ayurveda* gives for proper regulation of gut functions (*Agni*) to reduce *Ama* provides avenues for early-stage management of MASLD through diet, lifestyle modifications and therapeutic interventions (Feng et al., 2017; Fujisaka et al., 2024; Sharma, 2024).

The distinctive conceptualization of liver biology in *Ayurveda* and other traditional medical systems of South and South-East Asia provides a unique logic for systems-based therapeutics to restore the body’s metabolic homeostasis. These traditions extensively use medicinal plants by leveraging their phytochemical diversity, to create a rational blend of pharmacological properties that modulate interconnected metabolic pathways; thereby promoting adaptive and dynamic metabolic homeostasis. In *Ayurveda*, this pharmaceutical logic is systematically articulated through the concepts of *Rasapanchaka* of *Dravyaguṇa* (Joshi et al., 2007; Rath et al., 2014; Thakur et al., 2022). While this approach contrasts with reductionist pharmacology, it is increasingly supported by systems biology and network pharmacology; and is essential for managing diseases like MASLD that are arising from systemic dysregulation involving inter-organ communication (Chandran et al., 2017; Thottappillil et al., 2024). Supplementary Table 1 provides a curated list of medicinal plants illustrating how *Ayurveda*-informed phenotypic determinants translate into the selection of pharmacologically coherent, multi-target interventions relevant to MASLD.

## 4 Transdisciplinary framework for MASLD management: what is the way forward?

**FIGURE 2:**
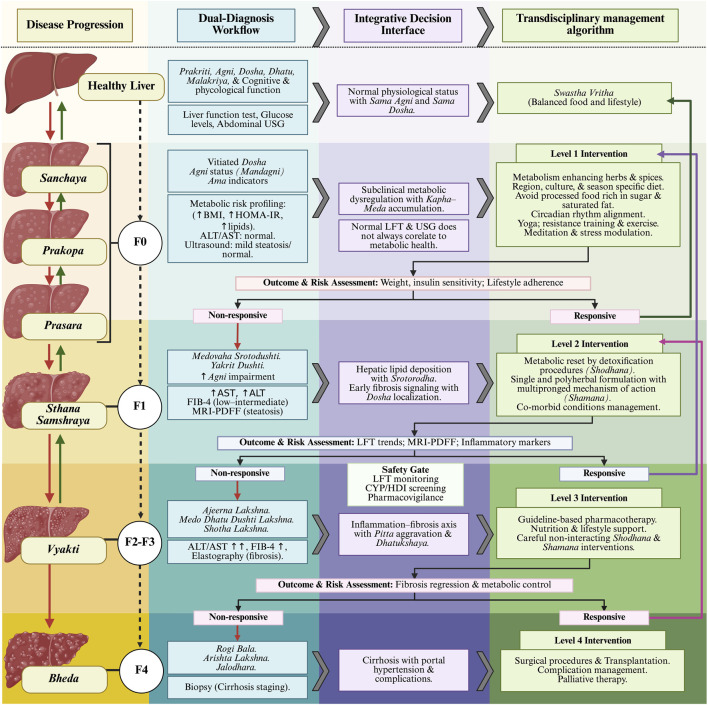
A unified transdisciplinary framework for stage-specific diagnosis and management of MASLD. Disease progression: Presents a hypothetical mapping of MASLD progression between Ayurvedic *Shadkriyakala* stages and biomedical fibrosis stages. Dual-diagnostic workflow: Presents a parallel diagnostic framework between *Ayurveda* and modern medicine. Diagnostic discordance between systems is anticipated and accommodated, with Ayurvedic assessment guiding process-oriented lifestyle and dietary interventions, and biomedical measures providing objective disease quantification and safety monitoring. Integrative decision interface: Provides a biologically plausible transdisciplinary decision interface arising from the dual-diagnostic workflow. Transdisciplinary management algorithm: Outlines a personalized, stage-specific therapeutic continuum, with early, reversible stages emphasizing *Ayurveda*-led nutrition, lifestyle, and mind–body interventions supported by biomedical monitoring. As disease progresses, therapeutic emphasis shifts toward integrated and subsequently biomedical-led strategies, with selective incorporation of traditional interventions within defined safety boundaries. Iterative feedback loops allow continuous reassessment, re-stratification, and escalation of care based on treatment response. ALT, Alanine aminotransferase; AST, Aspartate transaminase; USG, Ultrasound sonography; FIB-4, Fibrosis-4 index; MRI-PDFF, Magnetic resonance imaging proton density fat fraction; BMI, Body mass index; HOMA-IR, Homeostatic model assessment of insulin resistance; LFT, Liver function test; HDI, Herb-drug interaction; *Ajeerna lak* $\underset{˙}{s}$ *a* $\underset{˙}{n}$ *a* - Signs and symptoms of indigestion; *Medo Dhatu Du* $\underset{˙}{s}$ *h* $\underset{˙}{t}$ *i Lak* $\underset{˙}{s}$ *a* $\underset{˙}{n}$ *a*, Signs and symptoms of imbalance of *medo dhatu*; *Aris $\underset{˙}{t}$ a lak* $\underset{˙}{s}$ *a* $\underset{˙}{n}$ *a*, Prognostic signs of severe disease or poor vitality; *Jalodara*, Commonly understood as ascites; *Shotha Lak* $\underset{˙}{s}$ *a* $\underset{˙}{n}$ *a*, Signs and symptoms of swelling or edema; *Rogi Bala*, Resilience capacity of patient; *Malakriya*, excretory processes; *Dhatukshaya*, tissue depletion. Created in BioRender. Kouser, S. (2026) https://BioRender.com/mempdnx.

The interconnected tapestry of etio-pathophysiology of MASLD, governed by biological, lifestyle, environmental and sociocultural factors, demands an approach that transcends any single therapeutic framework. The very foundation of trans-disciplinarity is rooted in the principles of complexity science and chaos theory - disciplines that reveal an interconnected yet non-linear nature of reality. Human health and disease exemplify non-linear dynamic systems that require the integration of multiple, and often epistemologically distinct, knowledge systems (Jayasinghe, 2012; Biswas et al., 2018). The limitations of traditional lock-and-key framework in addressing the interconnectedness of complex etiopathological determinants highlight the need for systems approach and precision medicine models (Priego-Parra et al., 2025). Within this context, *Ayurveda*, as a whole-system medical tradition grounded in principles aligned with P4 medicine, offers a space for developing novel integrative strategies for MASLD management. Creating a transdisciplinary framework that bridges *Ayurveda* and conventional biomedicine opens new avenues for early detection, prevention, and personalized interventions to restore and maintain the dynamic metabolic homeostasis (Figure 2). The primary goal of this framework should be to prevent disease onset and progression of MASLD while improving the individual and population level quality of life through personalized, culturally-sensitive, and evidence-based strategies.

### 4.1 Dual-diagnostic workflow: integrating phenotyping and molecular pathology

Effective MASLD management requires early detection and personalization. However, no single diagnostic system adequately captures both metabolic vulnerability and structural pathology of the liver. To overcome this, a transdisciplinary dual-diagnostic framework, integrating *Ayurveda*-based phenotyping with established biomarkers would guide an integrative decision-making interface across the disease continuum (Figure 2). In this model, the readouts like molecular markers, fibrosis indices and imaging results provide objective quantification of hepatic pathophysiology and disease staging. Whereas *Ayurveda*-based diagnosis like *roga–rogi pareeksha* (disease and patient assessment) and other physiological assessments (*prakriti*, *vikriti*, *agni*, *ama*, and *srotodushti*) provides a systems-level abstraction of metabolic dysregulations arising from lifestyle and psychosomatic imbalance; which may precede overt biochemical or radiological readouts. These *Ayurveda*-based phenotyping can be leveraged to individualize lifestyle, dietary, circadian, and mind–body interventions for early stage management of MASLD.

### 4.2 Operationalizing transdisciplinary disease management algorithm

Diagnostic discordance between epistemologically distinct medical systems is anticipated and must be systematically addressed at the translational decision-making layer. Figure 2 proposes a four-level transdisciplinary management algorithm based on an integrative decision making framework. For example, individuals who have normal biomedical readouts but disturbed metabolic homeostasis on *Ayurveda* assessment are prioritized for preventive, low-risk lifestyle and dietary interventions (Level - 1), following integrative nutrition strategies combining principles of modern and *Ayurveda* dietetics. *Ayurveda* dietetics incorporates circadian and circannual rhythms, along with regional, cultural, and ecological determinants of food intake (Banerjee et al., 2015). When integrated with principles of food chemistry, nutrient bioavailability, and metabolic biochemistry it enables a novel framework that supports individualized dietary prescriptions tailored to ethnicity, nativity, and socioeconomic context. Beyond nutrition, the regulation of metabolic homeostasis in MASLD is increasingly recognized as a systems-level phenomenon governed by the psycho–neuro–endocrine–immunological (PNEI) network and the body–mind axis. Ayurvedic lifestyle practices such as Yoga, meditation, and structured physical activity are positioned as biologically relevant modulators of PNEI dynamics, with the potential to mitigate disease progression and enhance long-term metabolic resilience (González-Díaz et al., 2017).

As the disease progresses, the absence of liver-specific pharmacotherapies remains a major clinical challenge. In a transdisciplinary framework, the Ayurvedic assessment of the metabolic status of the disease through systematic evaluation of vitiated *Doshas*, affected *Dhatus* and *Agni* function can inform about the status of metabolic resilience, detoxification capacity, and tolerance to therapeutic load, which are particularly critical in the context of compromised hepatic drug metabolism (Kaur et al., 2024; Jayasuriya et al., 2025). In parallel, biomedical indicators including liver enzyme trends, quantitative steatosis, and glycaemic control provide objective measures of disease severity and physiological constraint. Together these insights are operationalized at the translational decision-making layer to modify therapeutic strategies that include evidence-based, well-tolerated *Shodhana* and *Shamana* modalities with minimal herb–drug interaction potential (Supplementary Table 1). At the advanced stages of MASLD with progressive inflammation, fibrosis, aggravated *Dosha* (*Pitta*) and *Dhatukshaya*, the treatment protocol shifts to guideline-based pharmacotherapy like Resmeritrom and careful non-interacting *Shamana* and *Shodhana* interventions; along with personalized nutrition and lifestyle support.

In the terminal-stage of disease, including decompensated cirrhosis and reduced quality of life, bariatric surgery and liver transplantation remain the cornerstone of management. However, integrative peri-operative care guided by biomedical surgical risk stratification and Ayurvedic functional assessment can support recovery through targeted nutritional optimization, functional conditioning, and stress modulation. These interventions are explicitly positioned as supportive and rehabilitative, not disease-modifying, and are aligned with standard-of-care clinical pathways (Figure 2).

Both biomedical and Ayurvedic systems converge on diet and lifestyle modification as foundational strategies for metabolic health. The transdisciplinary framework proposes the integration of *Ayurveda*’s seasonal and regional dietary guidance, informed by *prakriti* based body–mind phenotyping and *agni* assessment, as upstream strategies for personalized caloric moderation and whole-food dietary patterns. When systematically integrated into the public health nutrition policies, these principles can be combined with scalable interventions such as structured physical activity and mind–body practices such as yoga, enabling the operationalization of individualized metabolic health concepts into population-level preventive and promotive health strategies.

### 4.3 Challenges in operationalizing a transdisciplinary framework

Operationalizing a transdisciplinary framework necessitates the collaboration of teams proficient in both biomedical and traditional systems of medicine with a shared decision making algorithm supported by rigorous phytochemical standardization, GMP compliance for herbal products, robust pharmacovigilance frameworks to monitor potential drug–herb interactions, and the development of research infrastructure for personalization-focused *Ayurveda* clinical trials, multi-omics profiling, and computational biology. These measures are essential to transform integrative medicine from a co-practice model to a transdisciplinary paradigm for MASLD management ensuring safety, reproducibility, and clinical credibility.

#### 4.3.1 Quality control of drugs and pharmacovigilance

A central prerequisite for the successful clinical translation of any integrative or transdisciplinary framework involving herbal interventions is rigorous material characterization and standardization. The reproducibility crisis in herbal and traditional medicine research arises primarily from inadequate quality control and batch-to-batch variability of study materials; and therefore adherence to pharmacognostic and phytochemical standards is mandatory (Tandon, 2018; Tandon and Sharma, 2019). Comprehensive phytochemical profiling using state-of-the-art chromatographic fingerprinting methods and marker based standardizations following international standards like Consensus on Phytochemical Markers (ConPhyMP), to ensure botanical identity, chemical consistency, and traceability are advisable (Heinrich et al., 2022). While these are considered as a minimum requirement, the real need of the hour is to develop novel multi-marker or chemometrics approaches to capture the complexity of polyherbal preparations. Additionally, the Good Manufacturing Practice (GMP) compliance and robust pharmacovigilance mechanism to monitor potential herb–drug interactions are indispensable to ensure patient safety (Wal et al., 2011; Shaw et al., 2012). Perhaps pharmacovigilance is the most important aspect wherein hepatic drug-metabolizing capacity is drastically impaired in MASLD causing herb–drug interactions (HDIs) as a major clinical risk, potentially leading to herb-induced liver injury (HILI) (Volak et al., 2008; Al-Jenoobi et al., 2014; Ma et al., 2023; Somabattini et al., 2024). The proposed framework explicitly rejects the lay-man notion that “natural products are safe” and strongly argues for embedding phytochemical standardization and quality control as non-negotiable methodological requirements; enabling the framework to move beyond ‘philosophical hypothesis’ to a ‘pharmacologically and clinically accountable model’.

#### 4.3.2 Regulatory harmonization and ethical considerations

The clinical implementation of a transdisciplinary framework integrating Ayurvedic and biomedical interventions requires careful navigation of substantial regulatory and ethical complexities (Dubale et al., 2025). Across jurisdictions, traditional medicines and conventional pharmaceuticals are governed under distinct legal frameworks, creating asymmetries in approval pathways, quality oversight, and liability attribution (Chaudhary and Singh, 2011; Thamizhoviya, 2025; Tillu, 2025). For example, in India, Ayurvedic formulations are regulated under the Ministry of AYUSH, whereas biomedical drugs fall under the purview of the Central Drugs Standard Control Organization (CDSCO). In contrast, in many Western countries, Ayurvedic products are frequently marketed as dietary supplements with variable regulatory oversight. Such divergence raises critical questions regarding clinical responsibility and liability, particularly in the event of adverse outcomes arising from combined use. Here again, the article argues against any unsupervised or ad-hoc integration but emphasizes the need of regulatory harmonization through formally endorsed *Integrative Treatment Guidelines*. These guidelines should be ideally co-developed by regulatory authorities, professional medical councils, and traditional medicine bodies by clearly defining permissible combinations, safety requirements, documentation standards, and accountability structures for integrative care in MASLD. Furthermore, from an ethics point-of-view, the patients must be clearly informed about the rationale for integrative decision-making, the nature of the interventions, the existing level of evidence, and potential risks including herb–drug interactions; which will make the transdisciplinary framework a more disciplined, accountable, and patient-centred strategy that can evolve as a health policy (Gatt et al., 2024).

## 5 Conclusion

The global trend of traditional medicine-based disease management, particularly noncommunicable diseases, highlighted in the WHO’s Traditional Medicine Global Summit report (2023) (World Health Organization, 2023) underscores the need of harnessing the holistic understanding of the disease pathophysiology of the TMs with the mainstream treatment protocols. The proposed framework for MASLD management, integrating systemic insights of *Ayurveda* with molecular precision of biomedicine, offers promising early and preventive interventions, culturally aligned and personalized care, and multi-targeted therapeutics. However, the heterogeneity of herbal products, regulatory divergence across jurisdictions, limited mechanistic understanding of multi-targeted therapeutics, and the need for clinician cross-training remain as challenges. Overcoming them through coordinated education, research, and policy initiatives can open up novel integrative, globally adaptive and patient-centred management strategies for MASLD.

## 6 Future directions

For advancing the proposed transdisciplinary Ayurveda–Biology framework from a conceptual note to clinical implementation, the following priority areas warrant focused attention.

1. Standardization and Material Reproducibility: Rigorous phytochemical characterization and batch-to-batch standardization of Ayurvedic interventions using validated analytical platforms to ensure reproducibility, safety, and translational credibility.
2. Systems-Level Mechanistic Investigations: Development of innovative *in vitro*, *in vivo* and *in silico* model systems, employing transcriptomics, metabolomics, lipidomics, and network pharmacology, to capture the multi-component-multi-target mode of action of traditional formulations.
3. Robust Translational Decision Algorithms: Development of a carefully curated and scientifically validated translational framework to enable unified clinical decision-making rather than parallel practice.
4. Adaptive and Pragmatic Clinical Trial Designs: Establishing real-world evidence studies and personalization-focused clinical trials along with higher dimensional statistical analysis for overcoming the limitations of conventional randomized controlled trials.
5. Safety, Regulatory Guidelines and Capacity Building: Development of curated HDI databases, pharmacovigilance platforms, and unified regulatory guidelines for integrative treatments, endorsed by regulatory bodies. Providing structured training programs that equip clinicians and researchers with cross-disciplinary literacy for successful and sustainable implementation of a transdisciplinary framework.

## 7 Glossary of key *Ayurveda* terminologies used in the article

*Agni:* The simple translation of *Agni* is ‘fire’. However, in *Ayurveda*, *Agni* is a phenomenon that can be understood as a fundamental ‘energy form’ and governing principle of the biotransformation of substances in the body. At a gross level of physiology, *Agni* encompasses all the functions regulating digestion and absorption of nutrients from ingested food. At cellular and tissue level, *Agni* denotes the collective molecular pathways involved in biotransformation of nutrients to produce tissues (*dhatus)*, and energy. *Agni* is also responsible for catabolic activities. There are 13 types of *Agni* described in Ayurveda.

*Agni-mandya:* Refers to inappropriate functioning of *Agni* which eventually affects the digestion, absorption and assimilation of nutrients in *the* body.

*Ahara*: Food, drinks and other dietary substances consumed by the living organisms.

*Ahara Rasa:* The primary nutritive essence, generated after the digestion of food and drinks, that can be absorbed and ideally assimilated into the body. It supports seven tissue systems (*dhatus-* further explained) of the body.

*Ama:* It is used to denote the products and process of incomplete or inappropriate biotransformation of the nutrients at gastrointestinal tract and tissue levels. It also includes impaired products such as immunogenic *precursors* or aberrant metabolic intermediates of cellular biochemical transformation that eventually alter the normal biological functions. *Ama* formation is happening when the *Agni* functions are not proper. *Ama* is eliminated and further, its formation is prevented by modulating *Agni* through various methods including the medicinal herbs and formulations.

*Dhatu* (seven types): From *Ayurveda* epistemology, *Dhatu* denotes the fundamental building blocks of the body (roughly equivalent to the tissues in modern biology). There are seven *Dhatus* sequentially named as *Rasa* (∼ nutrient rich chyme)*, Rakta* (∼blood)*, Mamsa* (∼muscle)*, Meda* (∼ adipose tissue)*, Asthi* (∼ skeletal tissue)*, Majja* (∼ bone marrow) and *Sukra* (∼ reproductive tissue). *Rasa Dhatu* is the first *derivation* from ingested food and each of the subsequent *Dhatu* derives from the previous one in the sequence. However, each *dhatu* can be directly derived from *Ahara rasa.*

*Dhatu Agni:* Seven *Agni* are involved in the biotransformation of each *Dhatu* from its respective precursors. For e.g., The *Rasa Dhatu Agni* is responsible *for* conversion of *Ahara rasa* into *Rasa Dhatu*.

*Dravyaguna:* The Ayurvedic discipline that studies medicinal substances and explains their therapeutic actions based on their properties, actions, and interactions with the body.

*Prakriti:* Describes an individual’s inherent pattern of physical, physiological, and psychological traits, considered as a stable baseline that helps explain why people differ in metabolism, resilience, and health risks. *Prakriti* is defined as the independent or combined dominance of either *Vata*, *Pitta* or *Kapha Dosha*.

*Rasapanchaka:* The Ayurvedic framework describing drug action through five determinants referred as *rasa* (taste), *guna* (qualities), *virya* (potency), *vipaka* (post-digestive effect), and *prabhava* (specific action).

*Srotodushti:* Refers to the dysfunctions or disturbances in the body’s internal transport and communication pathways, known as *srotas*, which are responsible for the movement of nutrients, wastes, fluids, and signals throughout the body.

*Vikriti:* Denotes the “current state of imbalance” in a person’s body and mind, which *reflects* how lifestyle, diet, stress, environment, illness, or aging have temporarily disturbed the physiological homeostasis. *Vikriti* guides personalized corrective measures such as dietary changes, lifestyle adjustments, or treatments to restore health.

## Data Availability

The original contributions presented in the study are included in the article/Supplementary Material, further inquiries can be directed to the corresponding authors.

## References

[B1] Acharya Y. T. (1992a). Susrutha Samhitha with Dalhana Tika, Sharirasthana (9/12) (Varanasi: Chaukhambha Orientalia).

[B2] Acharya Y. T. (1992b). Susrutha Samhitha with Dalhana Tika, Sutrasthana (21/36–38) (Varanasi: Chaukhambha Orientalia).

[B3] Adeva-Andany M. M. Pérez-Felpete N. Fernández-Fernández C. Donapetry-García C. Pazos-García C. (2016). Liver glucose metabolism in humans. Biosci. Rep. 36, e00416. 10.1042/BSR20160385 27707936 PMC5293555

[B4] Adinolfi L. E. Marrone A. Izzi A. Craxì A. (2026). Management of metabolic dysfunction-associated steatotic liver disease (MASLD): facts and hopes. J. Clin. Exp. Hepatol. 16, 103411. 10.1016/J.JCEH.2025.103411 41480331 PMC12753517

[B5] Ahmed B. Sultana R. Greene M. W. (2021). Adipose tissue and insulin resistance in obese. Biomed. and Pharmacother. 137, 111315. 10.1016/J.BIOPHA.2021.111315 33561645

[B6] Al-Jenoobi F. I. Al-Thukair A. A. Alam M. A. Abbas F. A. Al-Mohizea A. M. Alkharfy K. M. (2014). Effect of Curcuma longa on CYP2D6- and CYP3A4-mediated metabolism of dextromethorphan in human liver microsomes and healthy human subjects. Eur. J. Drug Metabolism Pharmacokinet. 2014 40 (1), 61–66. 10.1007/S13318-014-0180-2 24510399

[B7] Aminian A. Al-Kurd A. Wilson R. Bena J. Fayazzadeh H. Singh T. (2021). Association of bariatric surgery with major adverse liver and cardiovascular outcomes in patients with biopsy-proven nonalcoholic steatohepatitis. JAMA - J. Am. Med. Assoc. 326, 2031–2042. 10.1001/JAMA.2021.19569 34762106 PMC8587225

[B8] Apte V. S. (1890). The practical Sanskrit-English dictionary, containing appendices on Sanskrit prosody and important literary and geographical names in the ancient history of India, for the use of schools and colleges. Poona, Shiralkar.

[B9] Arora C. Malhotra A. Ranjan P. Vikram N. K. Dwivedi S. N. Singh N. (2021). Perceived barriers and facilitators for adherence to lifestyle prescription: perspective of obese patients with non alcoholic fatty liver disease from North India. Diabetes and Metabolic Syndrome Clin. Res. and Rev. 15, 102138. 10.1016/J.DSX.2021.05.011 34186359

[B10] Asija R. Singh C. (2016). A comprehensive review on antihyperlipidemic activity of various medicinal plants. Int. J. Curr. Pharm. Rev. Res. 7. Available online at: www.ijcpr.com (Accessed January 16, 2026).

[B11] Azimi A. Jolfayi A. G. Rezayifar V. Ateen F. Fard H. H. Mohammed S. K. M. (2025). The association between metabolic-associated fatty liver diseases and risk of colorectal polyps, neoplasia, and cancer: a systematic review and meta-analysis of over 56 million individuals. Clin. Res. Hepatol. Gastroenterol. 49, 102652. 10.1016/j.clinre.2025.102652 40706955

[B12] Banerjee S. Debnath P. Debnath P. K. (2015). Ayurnutrigenomics: ayurveda-inspired personalized nutrition from inception to evidence. J. Tradit. Complement. Med. 5, 228–233. 10.1016/J.JTCME.2014.12.009 26587393 PMC4624353

[B13] Bhanji R. A. Narayanan P. Allen A. M. Malhi H. Watt K. D. (2017). Sarcopenia in hiding: the risk and consequence of underestimating muscle dysfunction in nonalcoholic steatohepatitis. Hepatology 66, 2055–2065. 10.1002/HEP.29420 28777879

[B14] Bilson J. Mantovani A. Byrne C. D. Targher G. (2024). Steatotic liver disease, MASLD and risk of chronic kidney disease. Diabetes Metab. 50, 101506. 10.1016/J.DIABET.2023.101506 38141808

[B15] Bishnoi N. Meena S. Sharma M. M. (2024). Kriyakala in ayurveda: understanding disease progression for precise treatments. J. Ayurveda Integr. Med. Sci. 9, 104–112. 10.21760/JAIMS.9.1.14

[B16] Biswas H. R. Hasan M. M. Kumar Bala S. (2018). Chaos theory and its applications in our real life. Barishal Univ. J. Part 1, 123–140.

[B17] Brauer M. Roth G. A. Aravkin A. Y. Zheng P. Abate K. H. Abate Y. H. (2024). Global burden and strength of evidence for 88 risk factors in 204 countries and 811 subnational locations, 1990–2021: a systematic analysis for the global burden of disease study 2021. Lancet 403, 2162–2203. 10.1016/S0140-6736(24)00933-4/ATTACHMENT/6C37FB42-9C2C-4C1C-8FCE-3D1A8278F6E8/MMC3.PDF 38762324 PMC11120204

[B18] Bril F. Lomonaco R. Orsak B. Ortiz-Lopez C. Webb A. Tio F. (2014). Relationship between disease severity, hyperinsulinemia, and impaired insulin clearance in patients with nonalcoholic steatohepatitis. Hepatology 59, 2178–2187. 10.1002/HEP.26988 24777953

[B19] Chandran U. Mehendale N. Patil S. Chaguturu R. Patwardhan B. (2017). Network pharmacology. *Innovative approaches in drug discovery: ethnopharmacology* . Syst. Biol. Holist. Target., 127–164. 10.1016/B978-0-12-801814-9.00005-2

[B20] Chaple J. Kolpakwar S. Prof A. (2016). Shatkriyakala-A novel concept for conservation of health.

[B21] Chaudhary A. Singh N. (2011). Contribution of world health organization in the global acceptance of ayurveda. J. Ayurveda Integr. Med. 2, 179–186. 10.4103/0975-9476.90769 22253507 PMC3255448

[B22] Chaudhuri S. Dutta A. Chakraborty S. B. D. (2023). Efficacy and safety of saroglitazar in real‐world patients of non‐alcoholic fatty liver disease with or without diabetes including compensated cirrhosis: a tertiary care center experience. JGH Open 7, 215–220. 10.1002/JGH3.12878 36968568 PMC10037031

[B23] Chauhan A. Semwal D. K. Mishra S. P. Semwal R. B. (2017). Ayurvedic concept of shatkriyakala: a traditional knowledge of cancer pathogenesis and therapy. J. Integr. Med. 15, 88–94. 10.1016/S2095-4964(17)60311-X 28285613

[B24] Chhabra M. Vidyasagar K. Gudi S. K. Sharma J. Sharma R. Rashid M. (2022). Efficacy and safety of saroglitazar for the management of dyslipidemia: a systematic review and meta-analysis of interventional studies. PLoS One 17, e0269531. 10.1371/journal.pone.0269531 35776741 PMC9249226

[B25] Cho C. H. Patel S. Rajbhandari P. (2023). Adipose tissue lipid metabolism: lipolysis. Curr. Opin. Genet. Dev. 83, 102114. 10.1016/J.GDE.2023.102114 37738733 PMC12834039

[B26] Cueto-Galán R. Fontalba-Navas A. Gutiérrez-Bedmar M. Ruiz-Canela M. Martínez-González M. A. Alves L. (2025). Adherence to the mediterranean diet to prevent or delay hepatic steatosis: a longitudinal analysis within the PREDIMED study. Front. Nutr. 12, 1518082. 10.3389/FNUT.2025.1518082 40469667 PMC12133482

[B27] Cuevas Garciá-Dorado S. Cornselsen L. Smith R. Walls H. (2019). Economic globalization, nutrition and health: a review of quantitative evidence. Glob. Health 15, 1–19. 10.1186/S12992-019-0456-Z/TABLES/2 PMC6381642 30786909

[B28] Dong T. Li J. Liu Y. Zhou S. Wei X. Hua H. (2024). Roles of immune dysregulation in MASLD. Biomed. and Pharmacother. 170, 116069. 10.1016/J.BIOPHA.2023.116069 38147736

[B29] Drygalski K. Drygalski K. (2025). Pharmacological treatment of MASLD: contemporary treatment and future perspectives. Int. J. Mol. Sci. 26, 26. 10.3390/IJMS26136518 PMC12249978 40650294

[B30] Dubale S. Usure R. E. Mekasha Y. T. Hasen G. Hafiz F. Kebebe D. (2025). Traditional herbal medicine legislative and regulatory framework: a cross-sectional quantitative study and archival review perspectives. Front. Pharmacol. 16, 1475297. 10.3389/FPHAR.2025.1475297/BIBTEX 39950109 PMC11821589

[B31] Eslam M. Sanyal A. J. George J. Sanyal A. Neuschwander-Tetri B. Tiribelli C. (2020). MAFLD: a consensus-driven proposed nomenclature for metabolic associated fatty liver disease. Gastroenterology 158, 1999–2014.e1. 10.1053/j.gastro.2019.11.312 32044314

[B32] Fakhry T. K. Mhaskar R. Schwitalla T. Muradova E. Gonzalvo J. P. Murr M. M. (2019). Bariatric surgery improves nonalcoholic fatty liver disease: a contemporary systematic review and meta-analysis. Surg. Obes. Relat. Dis. 15, 502–511. 10.1016/j.soard.2018.12.002 30683512

[B33] Feng Q. Liu W. Baker S. S. Li H. Chen C. Liu Q. (2017). Multi-targeting therapeutic mechanisms of the Chinese herbal medicine QHD in the treatment of non-alcoholic fatty liver disease. Oncotarget 8, 27820–27838. 10.18632/ONCOTARGET.15482 28416740 PMC5438611

[B34] Feng X. Zhang R. Yang Z. Zhang K. Xing J. (2024). Mechanism of metabolic Dysfunction-associated steatotic liver disease: important role of lipid metabolism. J. Clin. Transl. Hepatol. 12, 815–826. 10.14218/JCTH.2024.00019 39280069 PMC11393839

[B35] Formichi C. Caprio S. Nigi L. Dotta F. (2025). The impact of environmental pollution on metabolic health and the risk of non-communicable chronic metabolic diseases in humans. Nutr. Metabolism Cardiovasc. Dis. 35, 103975. 10.1016/J.NUMECD.2025.103975 40180824

[B36] Francque S. M. Bedossa P. Ratziu V. Anstee Q. M. Bugianesi E. Sanyal A. J. (2021). A randomized, controlled trial of the Pan-PPAR agonist lanifibranor in NASH. N. Engl. J. Med. 385, 1547–1558. 10.1056/NEJMOA2036205 34670042

[B37] Fujisaka S. Watanabe Y. Toume K. Morinaga Y. Nawaz A. Kado T. (2024). (2024). Identification of herbal drug extracts that promote growth of Akkermansia muciniphila in high-fat diet fed mice. Diabetol. Int. 15 (3), 495–506. 10.1007/S13340-024-00713-W 39101187 PMC11291798

[B38] Garg S. Varghese M. Shaik F. Jatin F. Sachdeva D. Eranhikkal F. W. (2025). Efficacy of non-invasive biomarkers in diagnosing non-alcoholic fatty liver disease (NAFLD) and predicting disease progression: a systematic review. Cureus 17, e78421. 10.7759/CUREUS.78421 40046382 PMC11881788

[B39] Gastaldelli A. Cusi K. (2019). From NASH to diabetes and from diabetes to NASH: mechanisms and treatment options. JHEP Rep. 1, 312–328. 10.1016/J.JHEPR.2019.07.002 32039382 PMC7001557

[B40] Gatt A. R. Vella Bonanno P. Zammit R. (2024). Ethical considerations in the regulation and use of herbal medicines in the European Union. Front. Med. Technol. 6, 1358956. 10.3389/FMEDT.2024.1358956 38948354 PMC11211540

[B41] González-Díaz S. N. Arias-Cruz A. Elizondo-Villarreal B. Monge-Ortega O. P. (2017). Psychoneuroimmunoendocrinology: clinical implications. World Allergy Organ. J. 10, 19. 10.1186/S40413-017-0151-6 28616124 PMC5460476

[B42] Harrison S. A. Bedossa P. Guy C. D. Schattenberg J. M. Loomba R. Taub R. (2024). A phase 3, randomized, controlled trial of resmetirom in NASH with liver fibrosis. N. Engl. J. Med. 390, 497–509. 10.1056/NEJMOA2309000 38324483

[B43] Heinrich M. Jalil B. Abdel-Tawab M. Echeverria J. Kulić Ž. McGaw L. J. (2022). Best practice in the chemical characterisation of extracts used in pharmacological and toxicological research—The ConPhyMP—Guidelines 12. Front. Pharmacol. 13, 953205. 10.3389/FPHAR.2022.953205/BIBTEX 36176427 PMC9514875

[B44] Hong X. Guo Z. Yu Q. (2023). Hepatic steatosis in women with polycystic ovary syndrome. BMC Endocr. Disord. 23, 1–11. 10.1186/S12902-023-01456-6/TABLES/8 37752440 PMC10521461

[B45] Hunter D. J. Reddy K. S. (2013). Noncommunicable. diseases. N. Engl. J. Med. 369, 1336–1343. 10.1056/NEJMra1109345 24088093

[B46] Hutchison A. L. Tavaglione F. Romeo S. Charlton M. (2023). Endocrine aspects of metabolic dysfunction-associated steatotic liver disease (MASLD): beyond insulin resistance. J. Hepatol. 79, 1524–1541. 10.1016/J.JHEP.2023.08.030/ASSET/7E4EEBFF-4723-4D27-810B-5867F200A9A3/MAIN.ASSETS/GR7.JPG 37730124

[B47] Issa G. Shang Y. Strandberg R. Hagström H. Wester A. (2025). Cause-specific mortality in 13,099 patients with metabolic dysfunction-associated steatotic liver disease in Sweden. J. Hepatol. 83, 643–651. 10.1016/j.jhep.2025.03.001 40139508

[B48] Jayasinghe S. (2012). Complexity science to conceptualize health and disease: is it relevant to clinical medicine? Mayo Clin. Proc. 87, 314–319. 10.1016/J.MAYOCP.2011.11.018 22469343 PMC3498395

[B49] Jayasuriya H. Kumar G. Sharma N. Sharma S. K. (2025). Medovaha srotodushti in non-alcoholic fatty liver disease: an integrated ayurvedic and contemporary perspective. Int. J. AYUSH.

[B50] Joshi K. Hankey A. Patwardhan B. (2007). Traditional phytochemistry: identification of drug by ‘taste’. Evidence-Based Complementary Altern. Med. 4, 145–148. 10.1093/ECAM/NEL064 17549231 PMC1876604

[B51] Kahn C. R. Wang G. Lee K. Y. (2019). Altered adipose tissue and adipocyte function in the pathogenesis of metabolic syndrome. J. Clin. Invest. 129, 3990–4000. 10.1172/JCI129187 31573548 PMC6763230

[B52] Kaur P. Dhankar S. Lata S. Kamath S. Sevatkar B. (2024). The critical appraisal of medovaha srotas: a comprehensive review of conceptual framework, clinical implications and contemporary perspective. J. Ayurveda Integr. Med. Sci. 9, 215–219. 10.21760/JAIMS.9.12.28

[B53] Kogiso T. Ogasawara Y. Taniai M. Tokushige K. Nakai Y. (2025). Extrahepatic events in patients with metabolic dysfunction-associated steatotic liver disease and the impact of genetics and alcohol intake. Hepatology Res. 55, 1335–1345. 10.1111/HEPR.14233 40590763

[B54] Lassailly G. Caiazzo R. Ntandja-Wandji L. C. Gnemmi V. Baud G. Verkindt H. (2020). Bariatric surgery provides long-term resolution of nonalcoholic steatohepatitis and regression of fibrosis. Gastroenterology 159, 1290–1301.e5. 10.1053/j.gastro.2020.06.006 32553765

[B55] Lawler P. R. Bhatt D. L. Godoy L. C. Lüscher T. F. Bonow R. O. Verma S. (2021). Targeting cardiovascular inflammation: next steps in clinical translation. Eur. Heart J. 42, 113–131. 10.1093/EURHEARTJ/EHAA099 32176778

[B56] Li X. He J. Sun Q. (2024). The prevalence and effects of sarcopenia in patients with metabolic dysfunction-associated steatotic liver disease (MASLD): a systematic review and meta-analysis. Clin. Nutr. 43, 2005–2016. 10.1016/J.CLNU.2024.07.006 39053329

[B57] Loomba R. Sanyal A. J. Kowdley K. V. Bhatt D. L. Alkhouri N. Frias J. P. (2023). Randomized, controlled trial of the FGF21 analogue pegozafermin in NASH. N. Engl. J. Med. 389, 998–1008. 10.1056/NEJMOA2304286 37356033 PMC10718287

[B58] Loomba R. Hartman M. L. Lawitz E. J. Vuppalanchi R. Boursier J. Bugianesi E. (2024). Tirzepatide for metabolic dysfunction–associated steatohepatitis with liver fibrosis. N. Engl. J. Med. 391, 299–310. 10.1056/NEJMOA2401943 38856224

[B59] Ludwig J. Viggiano T. R. McGill D. B. Ott B. J. (1980). Nonalcoholic steatohepatitis. Mayo clinic experiences with a hitherto unnamed disease. Mayo Clin. Proc. 55, 434–438. 10.1016/s0025-6196(24)00530-5 7382552

[B60] Luo L. Liu M. (2016). Adipose tissue in control of metabolism. J. Endocrinol. 231, R77. 10.1530/JOE-16-0211 27935822 PMC7928204

[B61] Ma Z. T. Shi Z. Xiao X. H. Wang J. B. (2023). New insights into herb-induced liver injury. Antioxid. Redox Signal. 38, 1138–1149. 10.1089/ARS.2022.0134;REQUESTEDJOURNAL:JOURNAL:AREA;WEBSITE:WEBSITE:SAGE;JOURNAL:JOURNAL:AREA;WGROUP:STRING:PUBLICATION 36401515 PMC10259609

[B62] Ma J. Ma Y. Wan X. Li J. Zhang Y. Liu J. (2025). Metabolic and genetic mechanisms of metabolic dysfunction-associated steatotic liver disease: an integrative perspective from molecular pathways to clinical challenges. Front. Endocrinol. (Lausanne) 16, 1639064. 10.3389/FENDO.2025.1639064/FULL 41001677 PMC12457186

[B63] Mahmood A. Nayak P. Deshmukh A. English C. N M. Solomon M. J. (2023). Measurement, determinants, barriers, and interventions for exercise adherence: a scoping review. J. Bodyw. Mov. Ther. 33, 95–105. 10.1016/J.JBMT.2022.09.014 36775533

[B64] Mallet M. Silaghi C. A. Sultanik P. Conti F. Rudler M. Ratziu V. (2024). Current challenges and future perspectives in treating patients with NAFLD-related cirrhosis. Hepatology 80, 1270–1290. 10.1097/HEP.0000000000000456 37183906

[B65] Mantovani A. Petracca G. Beatrice G. Csermely A. Lonardo A. Schattenberg J. M. (2022a). Non-alcoholic fatty liver disease and risk of incident chronic kidney disease: an updated meta-analysis. Gut 71, 156–162. 10.1136/GUTJNL-2020-323082 33303564

[B66] Mantovani A. Petracca G. Beatrice G. Csermely A. Tilg H. Byrne C. D. (2022b). Non-alcoholic fatty liver disease and increased risk of incident extrahepatic cancers: a meta-analysis of observational cohort studies. Gut 71, 778–788. 10.1136/GUTJNL-2021-324191 33685968

[B67] Merz K. E. Thurmond D. C. (2020). Role of skeletal muscle in insulin resistance and glucose uptake. Compr. Physiol. 10, 785–809. 10.1002/CPHY.C190029 32940941 PMC8074531

[B68] Mir B. A. Sharma B. Sharma R. Bodh V. Chauhan A. Majeed T. (2024). A prospective randomised comparative four-arm intervention study of efficacy and safety of saroglitazar and vitamin E in patients with non-alcoholic fatty liver disease (NAFLD)/Non-alcoholic steatohepatitis (NASH)-SVIN TRIAL. J. Clin. Exp. Hepatol. 14, 101398. 10.1016/j.jceh.2024.101398 38628977 PMC11017282

[B69] Mishra S. Bhujade H. Butt A. S. Kamani L. Premkumar M. (2022). Work-up for incidentally detected NAFLD: how far is it worth? Euroasian J. Hepatogastroenterol. 12, S26–S36. 10.5005/JP-JOURNALS-10018-1364 36466102 PMC9681574

[B70] Mishra S. Raj B. Kushwaha P. N. Verma D. Vijay M. (2025). Exploring the relationship between gut microbiota and agni: a comprehensive review. Vasc. and Endovascular Rev.

[B71] Mohan V. Joshi S. Kant S. Shaikh A. Sreenivasa Murthy L. Saboo B. (2025). Prevalence of metabolic dysfunction-associated steatotic liver disease: mapping across different Indian populations (MAP study). Diabetes Ther. 16, 1435–1450. 10.1007/S13300-025-01748-1 40381173 PMC12182534

[B72] Murthy S. R. K. (2001a). Astanga Hrdayam of Vagbhata. Sutrasthana (Varanasi: Krishnadas Academy), Vol. I, 11/33–34.

[B73] Murthy S. R. K. (2001b). Astanga Hrdayam of Vagbhata. Sutrasthana (Varanasi: Krishnadas Academy), Vol. I, 11/37–38.

[B74] Murthy S. R. K. (2001c). Astanga Hrdayam of Vagbhata. Sutrasthana (Varanasi: Krishnadas Academy), Vol. I, 12/11–12.

[B75] Murthy S. R. K. (ed.) (2001d). Astanga Hrdayam of Vagbhata, Vol. I, Sutrasthana (Chapter 13). Varanasi: Krishnadas Academy.

[B76] Musso G. Cassader M. Paschetta E. Gambino R. (2017). Thiazolidinediones and advanced liver fibrosis in nonalcoholic steatohepatitis: a meta-analysis. JAMA Intern. Med. 177, 633–640. 10.1001/JAMAINTERNMED.2016.9607 28241279 PMC5470366

[B77] Naaz A. Shivaprasad S. E. Patil A. S. (2025). Understanding relationship between concept of agni, ama and gut brain axis - contemporary review. J. Ayurveda Integr. Med. Sci. 10, 223–226. 10.21760/JAIMS.10.1.33

[B78] Nguyen M. H. Le M. H. Yeo Y. H. Zou B. Barnet S. Henry L. (2022). Forecasted 2040 global prevalence of nonalcoholic fatty liver disease using hierarchical bayesian approach. Clin. Mol. Hepatol. 28, 841–850. 10.3350/CMH.2022.0239 36117442 PMC9597215

[B79] Nowak K. Nowak A. Jabłońska A. Potaczek A. Salacha J. Dardzińska N. (2025). Implementation of noninvasive liver disease screening tools in primary care. Korean J. Fam. Med. 46, 381–390. 10.4082/KJFM.25.0144 41297559 PMC12661188

[B80] Portincasa P. Bonfrate L. Khalil M. De Angelis M. Calabrese F. M. D’amato M. (2021). Intestinal barrier and permeability in health, obesity and NAFLD. Biomed. 2022 10, 83. 10.3390/BIOMEDICINES10010083 35052763 PMC8773010

[B81] Prasad M. Sarin S. K. Chauhan V. (2023). Expanding public health responses to non-communicable diseases: the NAFLD model of India. Lancet Gastroenterol. Hepatol. 8, 969–970. 10.1016/S2468-1253(23)00312-6 37837972

[B82] Priego-Parra B. A. Gallego-Durán R. Román-Calleja B. M. Velarde-Ruiz Velasco J. A. Romero-Gómez M. Gracia-Sancho J. (2025). Advancing precision medicine in metabolic dysfunction-associated steatotic liver disease. Trends Endocrinol. Metabolism 36. 10.1016/j.tem.2025.03.006 40221323

[B83] Quek J. Chan K. E. Wong Z. Y. Tan C. Tan B. Lim W. H. (2023). Global prevalence of non-alcoholic fatty liver disease and non-alcoholic steatohepatitis in the overweight and obese population: a systematic review and meta-analysis. Lancet Gastroenterol. Hepatol. 8, 20–30. 10.1016/S2468-1253(22)00317-X 36400097

[B84] Rath S. Panja A. Nagar L. Shinde A. (2014). The scientific basis of rasa (taste) of a substance as a tool to explore its pharmacological behavior. Anc. Sci. Life 33, 198–202. 10.4103/0257-7941.147419 25593398 PMC4293745

[B85] Ray K. (2015). NAFLD. Leaky guts: intestinal permeability and NASH. Nat. Rev. Gastroenterol. Hepatol. 12, 123. 10.1038/NRGASTRO.2015.15;SUBJMETA 25645967

[B86] Riazi K. Azhari H. Charette J. H. Underwood F. E. King J. A. Afshar E. E. (2022). The prevalence and incidence of NAFLD worldwide: a systematic review and meta-analysis. Lancet Gastroenterol. Hepatol. 7, 851–861. 10.1016/S2468-1253(22)00165-0/ATTACHMENT/FF8825CF-F77C-4606-8D97-DD6F0EBEDD47/MMC1.PDF 35798021

[B87] Rinella M. E. Lazarus J. V. Ratziu V. Francque S. M. Sanyal A. J. Kanwal F. (2023). A multisociety Delphi consensus statement on new fatty liver disease nomenclature. J. Hepatol. 79, 1542–1556. 10.1016/j.jhep.2023.06.003 37364790

[B88] Rohm T. V. Meier D. T. Olefsky J. M. Donath M. Y. (2022). Inflammation in obesity, diabetes, and related disorders. Immunity 55, 31–55. 10.1016/J.IMMUNI.2021.12.013 35021057 PMC8773457

[B89] Saini P. Lata S. (2025). Ama: the hidden pathogen of Disease-A diagnostic insight. J. Emerg. Technol. Innov. Res. 12. Available online at: www.jetir.org (Accessed January 16, 2026).

[B90] Sanders F. W. B. Griffin J. L. (2015). *De novo* lipogenesis in the liver in health and disease: more than just a shunting yard for glucose. Biol. Rev. Camb. Philos. Soc. 91, 452–468. 10.1111/BRV.12178 25740151 PMC4832395

[B91] Sandireddy R. Sakthivel S. Gupta P. Behari J. Tripathi M. Singh B. K. (2024). Systemic impacts of metabolic dysfunction-associated steatotic liver disease (MASLD) and metabolic dysfunction-associated steatohepatitis (MASH) on heart, muscle, and kidney related diseases. Front. Cell Dev. Biol. 12, 1433857. 10.3389/FCELL.2024.1433857/XML 39086662 PMC11289778

[B92] Sanyal A. J. Newsome P. N. Kliers I. Østergaard L. H. Long M. T. Kjær M. S. (2025). Phase 3 trial of semaglutide in metabolic dysfunction–associated steatohepatitis. N. Engl. J. Med. 392, 2089–2099. 10.1056/NEJMOA2413258/SUPPL_FILE/NEJMOA2413258_DATA-SHARING.PDF 40305708

[B93] Sastry K. (1997a). Caraka Samhita of Agnivesa with Cakrapanidatta Tika, Part I, Sutrasthana (18/50–51) (Varanasi: Chaukhambha Sanskrit Sansthan).

[B94] Sastry K. (1997b). Caraka Samhita of Agnivesa with Cakrapanidatta Tika, Part I, Sutrasthana (28/4-5) (Varanasi: Chaukhambha Sanskrit Sansthan).

[B95] Sastry K. (1997c). Caraka Samhita of Agnivesa with Cakrapanidatta Tika, Part II, Chikitsa sthana (9/39 and 15/15) (Varanasi: Chaukhambha Sanskrit Sansthan).

[B96] Sastry K. (1997d). Caraka samhita of Agnivesa with cakrapanidatta tika, part II, Chikitsasthana (15/36) (Varanasi: Chaukhambha Sanskrit Sansthan).

[B97] Sastry K. (1997e). Caraka Samhita of Agnivesa with Cakrapanidatta Tika, Part II, Vimanasthana - Srotovimana Adhyaya (5/8-9) (Varanasi: Chaukhambha Sanskrit Sansthan).

[B98] Sastry K. (1997f). Caraka Samhita of Agnivesa with Cakrapanidatta Tika, Part II, Vimanasthana (5/8) (Varanasi: Chaukhambha Sanskrit Sansthan).

[B99] Schaffner F. Thaler H. (1986). Nonalcoholic fatty liver disease. Prog. Liver Dis. 8, 283–298. 3086934

[B100] Scoditti E. Sabatini S. Carli F. Gastaldelli A. (2024). Hepatic glucose metabolism in the steatotic liver. Nat. Rev. Gastroenterology and Hepatology 21 (5), 319–334. 10.1038/s41575-023-00888-8 38308003

[B101] Sharma M. (2024). Exploring the impact of virechan therapy on gut microbiota: insights from ayurveda. World J. Pharm. Med. Res., 2024. Available online at: https://www.wjpmr.com/download/article/123062024/1719664018.pdf (Accessed January 16, 2026).

[B102] Sharma P. Arora A. (2020). Clinical presentation of alcoholic liver disease and non-alcoholic fatty liver disease: spectrum and diagnosis. Transl. Gastroenterol. Hepatol. 5, 19. 10.21037/TGH.2019.10.02 32258523 PMC7063523

[B103] Shaw D. Graeme L. Pierre D. Elizabeth W. Kelvin C. (2012). Pharmacovigilance of herbal medicine. J. Ethnopharmacol. 140, 513–518. 10.1016/J.JEP.2012.01.051 22342381

[B104] Shruti Nandesh Mohan P. Hadapad H. M. (2025). Relation between agni and gut microbiota. J. Ayurveda Integr. Med. Sci. 10, 118–122. 10.21760/JAIMS.10.2.16

[B105] Somabattini R. A. Sherin S. Siva B. Chowdhury N. Nanjappan S. K. (2024). Unravelling the complexities of non-alcoholic steatohepatitis: the role of metabolism, transporters, and herb-drug interactions. Life Sci. 351, 122806. 10.1016/J.LFS.2024.122806 38852799

[B106] Steinberg G. R. Valvano C. M. De Nardo W. Watt M. J. (2025). Integrative metabolism in MASLD and MASH: pathophysiology and emerging mechanisms. J. Hepatol. 83, 584–595. 10.1016/j.jhep.2025.02.033 40032040

[B107] Tacke F. Horn P. Wai-Sun Wong V. Ratziu V. Bugianesi E. Francque S. (2024). EASL–EASD–EASO clinical practice guidelines on the management of metabolic dysfunction-associated steatotic liver disease (MASLD). J. Hepatol. 81, 492–542. 10.1016/J.JHEP.2024.04.031/ASSET/E92E5B46-7955-4358-85EC-34B4A3822047/MAIN.ASSETS/GR2.JPG 38851997

[B108] Tandon N. (2018). Phytochemical Reference Standards of Selected Indian Medicinal Plants (New Delhi: Indian Council of Medical Research).

[B109] Tandon N. Sharma P. (2019). Quality Standards of Indian Medicinal Plants. Indian Council of Medical Research. New Delhi: Indian Council of Medical Research.

[B110] Targher G. Byrne C. D. Tilg H. (2024). MASLD: a systemic metabolic disorder with cardiovascular and malignant complications. Gut 73, 691–702. 10.1136/GUTJNL-2023-330595 38228377

[B111] Thakur S. Sharma A. Sharma C. S. (2022). A Comparative study of Rasaand Phytochemicals of Dravyas-Literary Review. Int. J. Novel Res. Devel. 7 (12). Available online at: https://ijnrd.org/papers/IJNRD2212178.pdf (Accessed January 16, 2026).

[B112] Thamizhoviya G. (2025). Global integration of traditional and modern medicine: policy developments, regulatory frameworks, and clinical integration model. Future Integr. Med. 000, 180–190. 10.14218/FIM.2025.00033

[B113] Thottapillil A. Kouser S. Kukkupuni S. K. Vishnuprasad C. N. (2021). An ‘Ayurveda-Biology’ platform for integrative diabetes management. J. Ethnopharmacol. 268, 113575. 10.1016/j.jep.2020.113575 33181283

[B114] Thottappillil A. Sahoo S. Chakraborty A. Kouser S. Ravi V. Garawadmath S. (2024). *In vitro* and *in silico* analysis proving DPP4 inhibition and diabetes-associated gene network modulation by a polyherbal formulation: Nisakathakadi Kashaya. J. Biomol. Struct. Dyn. 42, 13588–13602. 10.1080/07391102.2023.2276880;CTYPE:STRING:JOURNAL 37938143

[B115] Tillu G. (2025). Ayush at crossroads - a challenge of opportunities. J. Ayurveda Integr. Med. 16, 101254. 10.1016/J.JAIM.2025.101254 40796456 PMC12368286

[B116] Trauner M. Arrese M. Wagner M. (2010). Fatty liver and lipotoxicity. Biochim. Biophys. Acta Mol. Cell Biol. Lipids 1801, 299–310. 10.1016/j.bbalip.2009.10.007 19857603

[B117] Tsofliou F. Vlachos D. Hughes C. Appleton K. M. Tsofliou F. Vlachos D. (2022). Barriers and facilitators associated with the adoption of and adherence to a mediterranean style diet in adults: a systematic review of published observational and qualitative studies. Nutr. 2022 14, 14. 10.3390/NU14204314 36296998 PMC9607475

[B118] Villanova N. Moscatiello S. Ramilli S. Bugianesi E. Magalotti D. Vanni E. (2005). Endothelial dysfunction and cardiovascular risk profile in nonalcoholic fatty liver disease. Hepatology 42, 473–480. 10.1002/HEP.20781 15981216

[B119] Volak L. P. Ghirmai S. Cashman J. R. Court M. H. (2008). Curcuminoids inhibit multiple human cytochromes P450, UDP- glucuronosyltransferase, and sulfotransferase enzymes, whereas piperine is a relatively selective CYP3A4 inhibitor. Drug Metabolism Dispos. 36, 1594–1605. 10.1124/dmd.108.020552 18480186 PMC2574793

[B120] Wal P. Wal A. Gupta S. Sharma G. Rai A. K. (2011). Pharmacovigilance of herbal products in India. J. Young Pharm. 3, 256–258. 10.4103/0975-1483.83780 21897669 PMC3159283

[B121] Wang L. Zhou H. Liu Y. Wang X. Yan W. Zhang J. (2023). Factors influencing adherence to lifestyle prescriptions among patients with nonalcoholic fatty liver disease: a qualitative study using the health action process approach framework. Front. Public Health 11, 1131827. 10.3389/FPUBH.2023.1131827/BIBTEX 37006574 PMC10065407

[B122] Willis L. H. Slentz C. A. Bateman L. A. Shields A. T. Piner L. W. Bales C. W. (2012). Effects of aerobic and/or resistance training on body mass and fat mass in overweight or obese adults. J. Appl. Physiol. 113, 1831–1837. 10.1152/JAPPLPHYSIOL.01370.2011 23019316 PMC3544497

[B123] World Health Organization (2014). WHO EMRO - global status reports. Available online at: https://www.emro.who.int/noncommunicable-diseases/publications/global-status-report-on-ncds.html (Accessed December 13, 2025).

[B124] World Health Organization (2023). WHO traditional medicine global summit 2023. Available online at: https://www.who.int/publications/m/item/who-traditional-medicine-global-summit-2023 (Accessed September 8, 2025).

[B125] World Health Organization (2025). Noncommunicable diseases. Available online at: https://www.who.int/news-room/fact-sheets/detail/noncommunicable-diseases (Accessed December 13, 2025).

[B126] Xu Q. Zhang J. Lu Y. Wu L. (2024). Association of metabolic-dysfunction associated steatotic liver disease with polycystic ovary syndrome. iScience 27, 108783. 10.1016/J.ISCI.2024.108783 38292434 PMC10825666

[B127] Younossi Z. M. Golabi P. Paik J. M. Henry A. Van Dongen C. Henry L. (2023). The global epidemiology of nonalcoholic fatty liver disease (NAFLD) and nonalcoholic steatohepatitis (NASH): a systematic review. Hepatology 77, 1335–1347. 10.1097/HEP.0000000000000004 36626630 PMC10026948

[B128] Younossi Z. M. Golabi P. Paik J. Owrangi S. Yilmaz Y. El-Kassas M. (2024a). Prevalence of metabolic dysfunction-associated steatotic liver disease in the Middle East and North Africa. Liver Int. 44, 1061–1070. 10.1111/LIV.15852 38305642

[B129] Younossi Z. M. Golabi P. Price J. K. Owrangi S. Gundu-Rao N. Satchi R. (2024b). The global epidemiology of nonalcoholic fatty liver disease and nonalcoholic steatohepatitis among patients with type 2 diabetes. Clin. Gastroenterology Hepatology 22, 1999–2010.e8. 10.1016/J.CGH.2024.03.006 38521116

[B130] Zargar A. H. Bhansali A. Majumdar A. Maheshwari A. Bhattacharyya A. Dasgupta A. (2025). Management of metabolic dysfunction–associated steatotic liver disease (MASLD)—An expert consensus statement from Indian diabetologists’ perspective. Diabetes Obes. Metab. 27, 3–20. 10.1111/DOM.16496 40457532 PMC12150358

