# Supplementary material for: A transdisciplinary framework for managing metabolic dysfunction associated steatotic liver disease
Source: Front Pharmacol. 2026 Feb 24;17:1767844. doi: 10.3389/fphar.2026.1767844 (PMC12971720; doi:10.3389/fphar.2026.1767844)
Supplement: Supplementary file 1 [file Table1.docx]

**Table 1.** Selected examples of commonly used plant drugs in management of *Yakrit roga*.

| Plant name | Scientific Name | Plant part used | *Ayurveda*-based characteristics of the plant | *Ayurveda*-based mode of action | Biomedicine-based mode of action | References |
| --- | --- | --- | --- | --- | --- | --- |
| *Bhumyamalaki* | *Phyllanthus amarus Schumach. & Thonn.* | Whole plant | *Katu*, *Tikta*, *Kashaya rasa*; *Laghu guna; Shita virya*. | Balances *Pitta* and *Kapha*. Liver detoxification and diuretic. | Improved insulin sensitivity and reduced hepatic oxidative stress and hyperlipidaemia. | (Adeneye, 2012; Sastry JLN, 2012; Putakala et al., 2017; Murthy KRS, 2021) |
| *Katuki* | *Picrorhiza kurroa*Royle ex Benth*.* | Root | *Tikta rasa*; *Laghu*, *Ruksha guna*; *Shita virya*. | Balances *Pitta* and *Kapha*. Powerful liver stimulant, purgative and digestive. | Anti-inflammatory and anti-fibrogenic effect, Increased mitochondrial biogenesis and reduced lipogenesis. | (Sastry JLN, 2012; Murthy KRS, 2021; Katoch et al., 2025) |
| *Punarnava* | *Boerhavia diffusa* L. | Root, leaves | *Madhura*, *Tikta*, *Kashaya* *rasa*; *Laghu*, *Ruksha guna*; *Ushna virya*. | Balances *Kapha* and *Vata*. Exhibits rejuvenation, diuretic and anti-inflammatory properties | Potent anti-obesity action | (Sastry JLN, 2012; Murthy KRS, 2021; Khalid et al., 2022) |
| *Patola* | *Trichosanthes dioica* Roxb. | Root | *Tikta* *rasa*; *Laghu*, *Ruksha guna*; *Ushna virya*. | Balances all three doshas. Liver detoxification and stimulant. Increases digestive ability. | Antihyperglycemic and antihyperlipidemic effect. | (Sastry JLN, 2012; Rai et al., 2013; Murthy KRS, 2021) |
| *Yashtimadhu* | *Glycyrrhiza glabra* L. | Root & stem | *Madhura* *rasa*; *Guru,* *Snigdha* *guna*; *Sheet virya*. | Balances *Vata* and *Pitta*. Rejuvenator and anti-inflammatory. | Hepatoprotective and anti-inflammatory effect. Reduced oxidative stress. | (Sastry JLN, 2012; Murthy KRS, 2021; Uyar et al., 2024; Alrefaei and Elbeeh, 2025) |
| *Amalaki* | *Phyllanthus emblica* L. | Fruit | *Amla*, *Madhura*, *Tikta*, *Kashaya*, *Katu* *rasa*; *Laghu*, *Ruksha guna*; *Shita virya*. | Balances all three *doshas*. Potent rejuvenator, antioxidant, immunomodulator, and digestive aid. | Anti-steatotic, anti-hyperlipidemic and antioxidant activity. Modulatory effect on gut microbiota and microbial metabolism. | (Sastry JLN, 2012; Huang et al., 2017; Murthy KRS, 2021; Luo et al., 2022) |
| *Haritaki* | *Terminalia chebula*Retz. | Fruit | *Kashaya*, *Katu*, *Amla,* *Madhura*, *Tikta* *rasa*; *Laghu*, *Ruksha guna*; *Ushna virya*. | Balances all three *doshas*. Potent rejuvenator, digestive aid and detoxification properties. | Anti-inflammatory and anti-diabetic activity. | (Sastry JLN, 2012; Murthy KRS, 2021; Agrawal and Kulkarni, 2023; El-Shamarka et al., 2024) |
| *Vibhitaki* | *Terminalia bellirica* (Gaertn.) Roxb. | Fruit | *Kashaya*,  *Madhura* *rasa*; *Laghu*, *Ruksha guna*; *Ushna virya*. | Balances *Pitta* and *Kapha*. Anti-inflammatory properties and digestive aid. | Anti-steatotic and anti-hyperlipidemic activity. Modulatory effect on gut microbiota. | (Sastry JLN, 2012; Murthy KRS, 2021; Zhang et al., 2023) |
| *Madhunashini* | *Gymnema sylvestre* (Retz.) R.Br. ex Sm. | Leaves | *Tikta*, *Kashaya* *rasa*; *Laghu*, *Ruksha guna*; *Ushna virya*. | Balances *Kapha* and *Vata*. Anti-diabetic, anti-inflammatory and digestive aid. | Anti-hyperglycemic and anti-hyperlipidemic effect. | (Sastry JLN, 2012; Murthy KRS, 2021; Muzaffar et al., 2023) |
| *Kiratatikta* | *Swertia chirayita* (Roxb.) H.Karst. | Whole plant | *Tikta* *rasa*; *Laghu*, *Ruksha guna*; *Shita virya*. | Balances *Pitta* and *Kapha.* Liver stimulant, digestive aid and anti-pyretic. | Antioxidant and anti-steatotic effect. | (Chen et al., 2011; Sastry JLN, 2012; Murthy KRS, 2021; Xue et al., 2024) |

Footnotes: *Amla rasa* (Sour), *Madhura* *rasa* (sweet), *Tikta* *rasa* (bitter), *Kashaya* *rasa* (astringent), *Katu* *rasa* (pungent), *Laghu* *guna* (light), *Ruksha guna* (dry), *Guru* *guna* (heavy), *Snigdha* *guna* (unctuous), *Sheet virya* (cooling) and *Ushna virya* (heating).

**References**

Adeneye, A. A. (2012). The leaf and seed aqueous extract of Phyllanthus amarus improves insulin resistance diabetes in experimental animal studies. *J. Ethnopharmacol.* 144, 705–711. doi: 10.1016/J.JEP.2012.10.017

Agrawal, O. D., and Kulkarni, Y. A. (2023). Treatment with Terminalia chebula Extract Reduces Insulin Resistance, Hyperglycemia and Improves SIRT1 Expression in Type 2 Diabetic Rats. *Life 2023, Vol. 13, Page 1168* 13, 1168. doi: 10.3390/LIFE13051168

Alrefaei, A. F., and Elbeeh, M. E. (2025). Hepatoprotective Effects of Glycyrrhiza glabra in Diabetic Male Rats: Addressing Liver Function, Oxidative Stress, and Histopathological Changes. *Biology 2025, Vol. 14, Page 307* 14, 307. doi: 10.3390/BIOLOGY14030307

Chen, Y., Huang, B., He, J., Han, L., Zhan, Y., and Wang, Y. (2011). In vitro and in vivo antioxidant effects of the ethanolic extract of Swertia chirayita. *J. Ethnopharmacol.* 136, 309–315. doi: 10.1016/J.JEP.2011.04.058

El-Shamarka, M. E. A., Aboulthana, W. M., Omar, N. I., and Mahfouz, M. M. (2024). Evaluation of the biological efficiency of Terminalia chebula fruit extract against neurochemical changes induced in brain of diabetic rats: an epigenetic study. *Inflammopharmacology 2024 32:2* 32, 1439–1460. doi: 10.1007/S10787-024-01428-9

Huang, C. Z., Tung, Y. T., Hsia, S. M., Wu, C. H., and Yen, G. C. (2017). The hepatoprotective effect of Phyllanthus emblica L. fruit on high fat diet-induced non-alcoholic fatty liver disease (NAFLD) in SD rats. *Food Funct.* 8, 842–850. doi: 10.1039/C6FO01585A

Katoch, S., Chhimwal, J., Singh, D., Kumar, D., and Patial, V. (2025). Picrosides-rich fraction from Picrorhiza kurroa attenuates steatohepatitis in zebrafish and mice by modulating lipid metabolism and inflammation. *Phytomedicine* 137, 156368. doi: 10.1016/J.PHYMED.2025.156368

Khalid, M., Alqarni, M. H., Shoaib, A., Wahab, S., Foudah, A. I., Aljarba, T. M., et al. (2022). Anti-Obesity Action of Boerhavia diffusa in Rats against High-Fat Diet-Induced Obesity by Blocking the Cannabinoid Receptors. *Plants 2022, Vol. 11, Page 1158* 11, 1158. doi: 10.3390/PLANTS11091158

Luo, X., Zhang, B., Pan, Y., Gu, J., Tan, R., and Gong, P. (2022). Phyllanthus emblica aqueous extract retards hepatic steatosis and fibrosis in NAFLD mice in association with the reshaping of intestinal microecology. *Front. Pharmacol.* 13, 893561. doi: 10.3389/FPHAR.2022.893561/BIBTEX

Murthy KRS (2021). *Bhavaprakasha of Bhavamishra*. Varanasi: Chaukhambha Krishnadas Academy.

Muzaffar, H., Qamar, I., Bashir, M., Jabeen, F., Irfan, S., and Anwar, H. (2023). Gymnema Sylvestre Supplementation Restores Normoglycemia, Corrects Dyslipidemia, and Transcriptionally Modulates Pancreatic and Hepatic Gene Expression in Alloxan-Induced Hyperglycemic Rats. *Metabolites* 13. doi: 10.3390/METABO13040516

Putakala, M., Gujjala, S., Nukala, S., and Desireddy, S. (2017). Beneficial Effects of Phyllanthus amarus Against High Fructose Diet Induced Insulin Resistance and Hepatic Oxidative Stress in Male Wistar Rats. *Applied Biochemistry and Biotechnology 2017 183:3* 183, 744–764. doi: 10.1007/S12010-017-2461-0

Rai, K., Gupta, S. K., Srivastava, A. K., Kumar Gupta, R., Watal, G., Miller, D. K., et al. (2013). A Scientific Validation of Antihyperglycemic and Antihyperlipidemic Attributes of Trichosanthes dioica. *Int. Sch. Res. Notices* 2013, 473059. doi: 10.1155/2013/473059

Sastry JLN (2012). *Dravyaguna Vijnana: Study of Medicinal Plants in Ayurveda*. Varanasi: Chaukhambha Orientalia.

Uyar, A., Özdere, B., Yaman, T., and Ufuk Kömüroğlu, A. (2024). Effects of licorice root (Glycyrrhiza glabra) extract on the livers of obese rats*. *Biotechnic and Histochemistry* 99, 370–378. doi: 10.1080/10520295.2024.2401159;PAGE:STRING:ARTICLE/CHAPTER

Xue, J., Zhang, L., Tao, J., Xie, X., Wang, X., Wu, L., et al. (2024). A novel bellidifolin intervention mitigates nonalcoholic fatty liver disease-like changes induced by bisphenol F. *J. Biomed. Res.* 38, 451–463. doi: 10.7555/JBR.37.20230169

Zhang, B., Luo, X., Han, C., Liu, J., Zhang, L., Qi, J., et al. (2023). Terminalia bellirica ethanol extract ameliorates nonalcoholic fatty liver disease in mice by amending the intestinal microbiota and faecal metabolites. *J. Ethnopharmacol.* 305, 116082. doi: 10.1016/J.JEP.2022.116082
